# Supplementary figures and images for: Non-Destructive Removal of Dental Implant by Using the Cryogenic Method
Source: Medicina (Kaunas). 2022 Jun 25;58(7):849. doi: 10.3390/medicina58070849 (PMC9319264; doi:10.3390/medicina58070849)

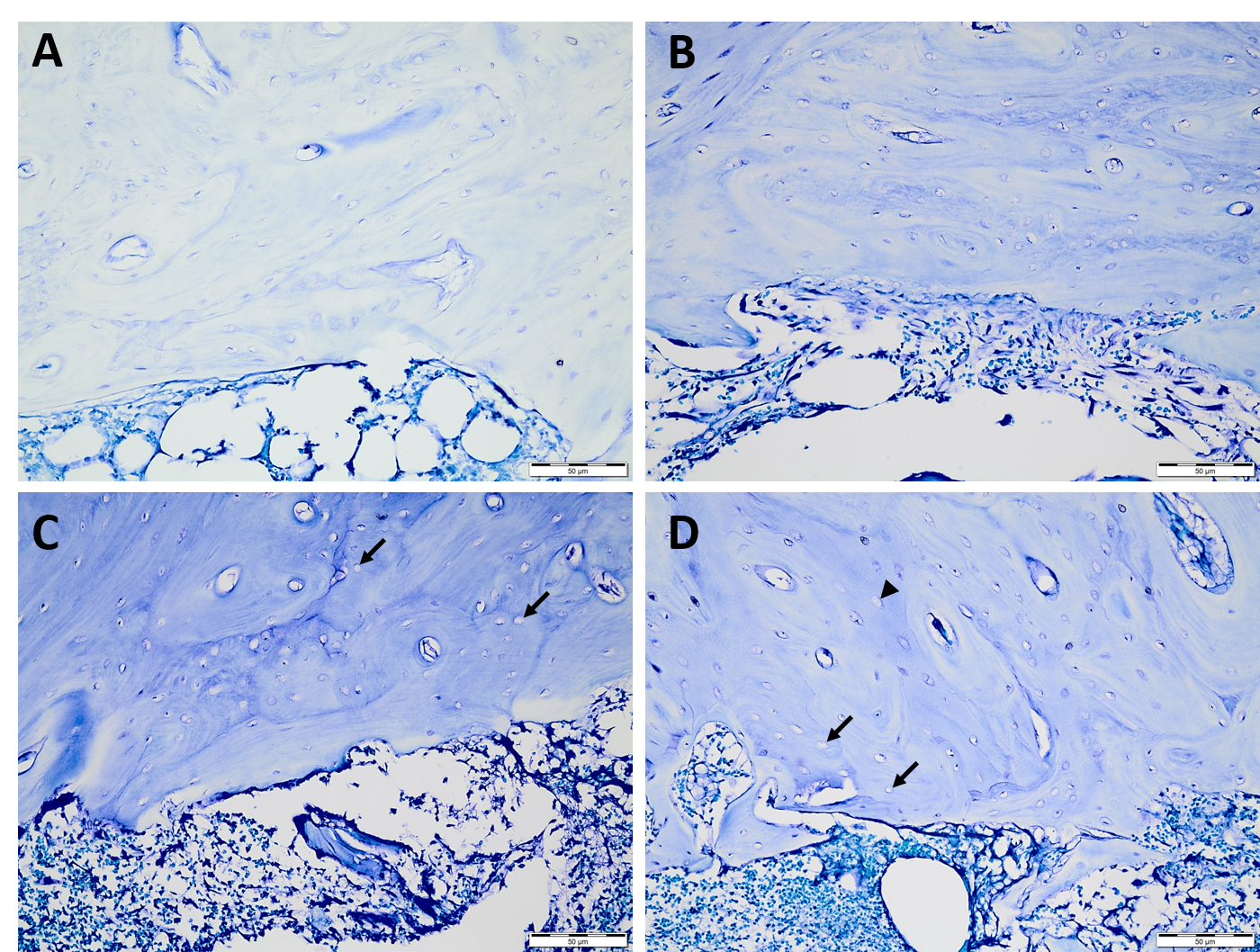

Supplement: Supplementary file 1 [file medicina-58-00849-s001.zip › Figure S1.tif]

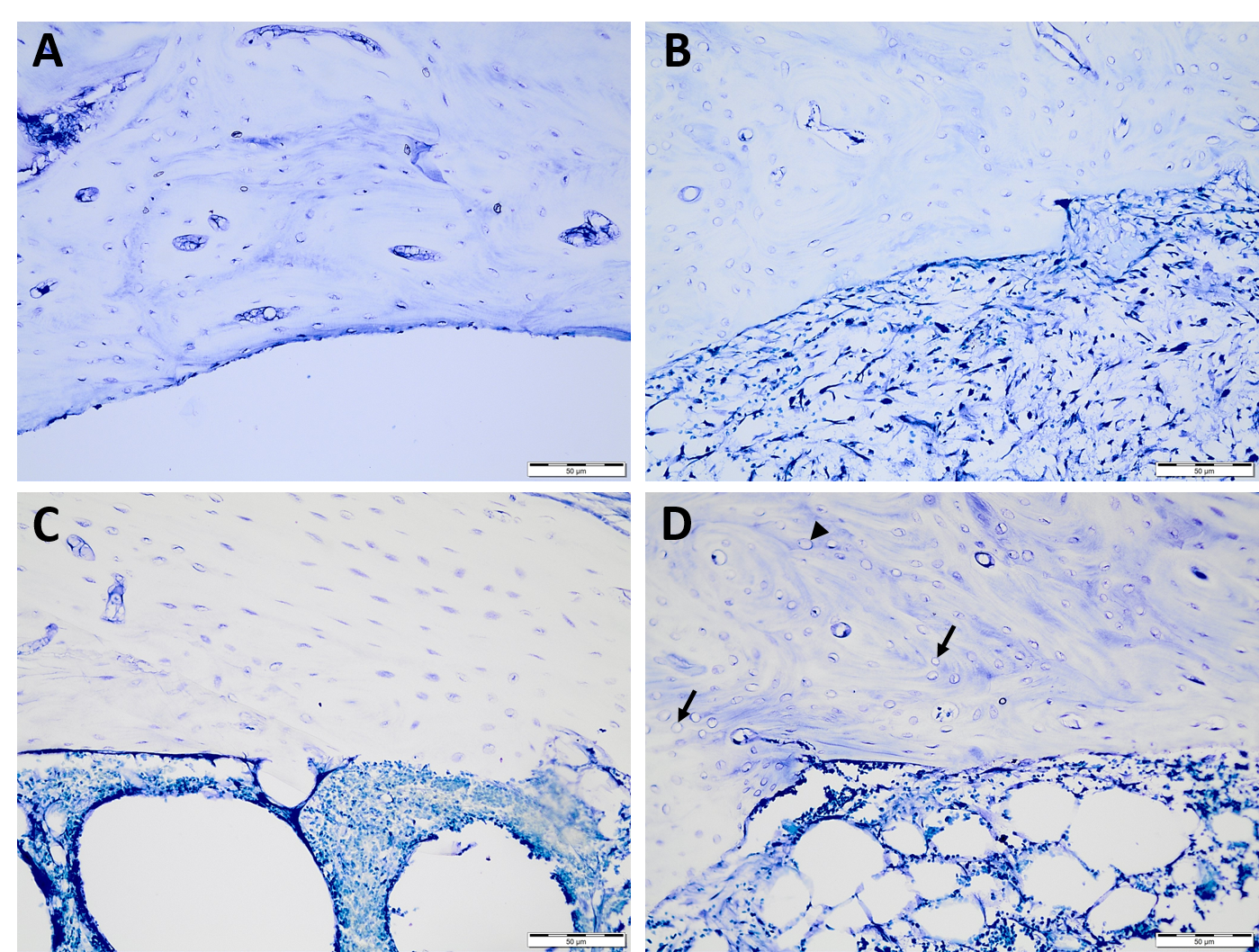

Supplement: Supplementary file 1 [file medicina-58-00849-s001.zip › Figure S2.tif]
